# *H. pylori* Related Atrophic Gastritis Detection Using Enhanced Convolution Neural Network (CNN) Learner

**DOI:** 10.3390/diagnostics13030336

**Published:** 2023-01-17

**Authors:** Yasmin Mohd Yacob, Hiam Alquran, Wan Azani Mustafa, Mohammed Alsalatie, Harsa Amylia Mat Sakim, Muhamad Safiih Lola

**Affiliations:** 1 Faculty of Electronic Engineering & Technology, Pauh Putra Campus, Universiti Malaysia Perlis (UniMAP), Arau 02600, Perlis, Malaysia; 2 Centre of Excellence for Advanced Computing, Pauh Putra Campus, Universiti Malaysia Perlis (UniMAP), Arau 02600, Perlis, Malaysia; 3 Department of Biomedical Systems and Informatics Engineering, Yarmouk University, Irbid 21163, Jordan; 4 Department of Biomedical Engineering, Jordan University of Science and Technology, Irbid 22110, Jordan; 5 Faculty of Electrical Engineering & Technology, Pauh Putra Campus, Universiti Malaysia Perlis (UniMAP), Arau 02600, Perlis, Malaysia; 6 King Hussein Medical Center, Royal Jordanian Medical Service, The Institute of Biomedical Technology, Amman 11855, Jordan; 7 School of Electrical and Electronic Engineering, Engineering Campus, Universiti Sains Malaysia, Nibong Tebal 11800, Penang, Malaysia; 8 Faculty of Ocean Engineering Technology and Informatics, Universiti Malaysia Terengganu, Kuala Terengganu 21030, Terengganu, Malaysia

**Keywords:** *H. pylori*, atrophic gastritis, deep learning, convolution neural network, ShuffleNet, feature fusion, Canonical Correlation Analysis, ReliefF, generalized additive model

## Abstract

Atrophic gastritis (AG) is commonly caused by the infection of the *Helicobacter pylori* (*H. pylori*) bacteria. If untreated, AG may develop into a chronic condition leading to gastric cancer, which is deemed to be the third primary cause of cancer-related deaths worldwide. Precursory detection of AG is crucial to avoid such cases. This work focuses on *H. pylori*-associated infection located at the gastric antrum, where the classification is of binary classes of normal versus atrophic gastritis. Existing work developed the Deep Convolution Neural Network (DCNN) of GoogLeNet with 22 layers of the pre-trained model. Another study employed GoogLeNet based on the Inception Module, fast and robust fuzzy C-means (FRFCM), and simple linear iterative clustering (SLIC) superpixel algorithms to identify gastric disease. GoogLeNet with Caffe framework and ResNet-50 are machine learners that detect *H. pylori* infection. Nonetheless, the accuracy may become abundant as the network depth increases. An upgrade to the current standards method is highly anticipated to avoid untreated and inaccurate diagnoses that may lead to chronic AG. The proposed work incorporates improved techniques revolving within DCNN with pooling as pre-trained models and channel shuffle to assist streams of information across feature channels to ease the training of networks for deeper CNN. In addition, Canonical Correlation Analysis (CCA) feature fusion method and ReliefF feature selection approaches are intended to revamp the combined techniques. CCA models the relationship between the two data sets of significant features generated by pre-trained ShuffleNet. ReliefF reduces and selects essential features from CCA and is classified using the Generalized Additive Model (GAM). It is believed the extended work is justified with a 98.2% testing accuracy reading, thus providing an accurate diagnosis of normal versus atrophic gastritis.

## 1. Introduction

Artificial intelligence (AI) has been important in steering various medical imaging-related problems. Although consultancy or diagnosis from medical practitioners has been the gold standard, computer-aided systems play a key role in assisting the limited number of busy-scheduled medical practitioners. Moreover, enhancing medical imaging techniques via AI is useful for screenings, medical precision, and risk assessment.

Otherwise, AI implementation in medical imaging enables physicians to detect medical-related conditions faster, thus promoting early intervention. AI enables colorectal cancer to be detected and diagnosed by analyzing tissue scans [1]. Furthermore, AI can identify fractures [2], neurological diseases [3], and thoracic complications [4]. Recent works have highlighted that AI may be better or at least on par with pathologists. It is observed that AI can be a tool to assist pathologists in keeping up with the rising demands for expert services. Despite pathologists regularly assessing histopathology images, their average workload has risen significantly, leading to unintended misdiagnosis [5]. The fusion of AI into medical imaging also enhances precision medicine. Outside of this, a machine learning tool can recognize types of cancer [6] and predict patient survival rates, resulting in healthcare providing better treatment [7]. In addition, AI can be a tool to predict potential risks for the future, such as the prediction of heart attacks [8].

Various studies that employ machine learning are also significant for sparking further applied or improved works. Weis et al. developed unsupervised segmentation via Linear Combination [9]. Beneficial works are segmentation in medical imaging [10,11] and studies related to body parts [12,13] that employed deep learning. Skin-lesion segmentation implemented using hybrid metaheuristics machine learner has also been studied [14]. Lv et al. highlighted speech and gesture recognition and natural language processing studies implemented using deep learning [15]. Face image identification has been used to detect fake faces via Xception, which is a variation of deep learning [16]. There has also been work on disease diagnosis implemented using Convolution Neural Network (CNN) [17,18]. Recognition of different types of skin lesions using Fast Random Forest (FRF) learner has been beneficial [19]. Massaro et al. performed comparative studies via Discrete Fourier Transform (DCT), K-Means clustering, and Long-Short Term Memory (LSTM) to determine assembled tires defects [20]. Mohimont et al. estimated crop yielding via machine learning and deep learning [21]. Massaro et al. identified water leaks in pipelines using infrared thermography (IRT), CNN-LSTM, and image filtering algorithm [22].

Similarly, AI-based research impacts significantly on recognizing internal gastric infection. Untreated *Helicobacter pylori* (*H. pylori*) gastric bacterial infection may lead to gastric cancer, among other complex morbidities. AG, which is mostly caused by *H. pylori* bacteria, induces chronic inflammation of gastric mucosa leading to the loss of glands in the stomach lining. Additionally, *H. pylori*-associated AG increases the risk of developing stomach cancer. Therefore, preliminary detection of AG is crucial.

To date, many meta-analyses have focused on the application of AI-based *H. pylori* diagnosis [23,24,25] and gastric disease [26,27]. Five studies [28,29,30,31,32] developed an AI algorithm based on Convolution Neural Network (CNN) to diagnose *H. pylori* infection. Studies [33,34] have employed AI to diagnose gastric disease and deep Recurrent Neural Network (RCNN) to diagnose AG progression to gastric cancer [35]. In addition, Support Vector Machine (SVM) algorithms have also been employed to recognize gastric disease [36,37] and *H. pylori* infection in the gut [38]. Yet another work with biomarkers features has used statistical analysis to detect AG [39].

This work acknowledged Pecere et al.’s [25] critique that many studies focus on selected populations that may experience spectrum bias or class imbalance. Pecere et al. [25] argued some studies have tendencies to employ CNN regardless of other available machine learners mentioned in Mohan et al. [24]. To date, the pooled accuracy, sensitivity, and specificity of AI in the diagnosis of gastrointestinal disease is 87.1% (95% CI 81.8–91.1), 86.3% (95% CI 80.4–90.6), and 87.1% (95% CI 80.5–91.7), respectively [25]. The recognition rates of the three measures indicated were between 86.3% and 87.1%, demonstrating an improvement in AI-based AG-related detection. This is still acceptable even when some classifiers, such CNN, do not satisfy the recognition rates in research connected to AG.

The essential issue is to find a better machine learner to improve the standard of diagnostic results. Hence, an accurate, fast, and reliable diagnosis is needed to treat *H. pylori* infection early. This is important in bacteria transmission inhibition, which promotes a longer lifespan.

## 2. Related Works

Recent trends have a growing interest in incorporating artificial intelligence (AI), medical diagnosis, and medical imaging related to the area in gastric. Getting a correct diagnosis with the assistance of a machine learner leads to a positive response because it is consistent and less likely to have inter-reader variability. Similar to other medical conditions, early detection of *Helicobacter pylori* (*H. pylori*) infection in human gastric is essential to acquire treatment and suppress the progression of this bacteria. Note that *H. pylori* cause not only gastritis, but untreated infections may also turn into chronic gastritis that leads to gastro-duodenal ulcer, gastric neoplasia, and gastric cancer [24].

This work highlights AI-based studies to detect *H. pylori*-related AG and other types of conditions in the gut. For example, Takiyama et al. [40] and Wu et al. [41] both examined gastric cancer, particularly to identify the anatomic multi-location of cancer. Wu et al. found an accuracy of 90% for detecting gastric cancer location-wise, and Takiyama showed an Area Under Curve (AUC) of 0.99, a sensitivity of 96.9%, and a specificity of 98.5%, respectively. On the other hand, Kanesaka et al. [37] detected 66 abnormal conditions out of 126 images during training and 61 abnormalities out of 81 images during testing. Furthermore, the study employed a Support Vector Machine (SVM) to detect early gastric cancer with an accuracy of 96.3%, a sensitivity of 96.7%, and a specificity of 95%. This result is quite impressive compared to works that employed CNN that mostly report around 85% to 93.8% accuracy.

In 2018, Hirasawa et al. [42] identified gastric cancer using Single Shot MultiBox Detector (SSD) with a sensitivity of 92.2% via 13,584 abnormalities during training and 2.296 abnormalities during testing out of 51,558 images. Another work by Ishioka et al. [43] also detected gastric cancer via SSD with a sensitivity of 94.1%. However, unlike Li et al. [44], Horuichi et al. [45] and Zhu et al. [46] both managed to detect gastric cancer with an accuracy of 85.3% and 89.16%, respectively. Meanwhile, Li et al. scored 90.91% accuracy in identifying gastric cancer via CNN Inception-V3. The other two studies also employed CNN, GoogLeNet, and ResNet, respectively.

Subsequent works describe studies related to machine learning detecting *H. pylori* infection in the gastric antrum. For example, Shichijo et al. employed Deep CNN (DCNN) of GoogLeNet with 22 layers of the pre-trained model that learned generic image features of *H. pylori*-infected cases. The AUC was 0.89, while sensitivity, specificity, and accuracy were 81.9%, 83.4%, and 83.1%, respectively [28]. Otherwise, Shichijo et al. collected a huge range of data with 32,208 images; 735 were *H. pylori* positive and 1015 were negative [28]. Another work by Shichijo et al. in 2019 [31] recorded an 80% accuracy (465/582) of negative diagnoses, 84% (147/174) eradication, and 48% (44/91) positive diagnoses. Similarly, the author employed a deep pre-trained CNN learner to identify the occurrence of *H. pylori* in the gastric antrum.

Alternatively, Itoh et al. [29] and Dong-Hyun et al. [33] both employed GoogLeNet to detect *H. pylori* conditions and gastric lesions, respectively. Dong-Hyun et al. employed GoogLeNet based on the Inception module, fast and robust fuzzy C-means (FRFCM), and simple linear iterative clustering (SLIC) superpixel algorithms applied for image segmentation during preprocessing of those images. Their work scored 0.85 and 0.87 for normal and abnormal lesions based on Receiver Operating Characteristic (ROC) curves. Meanwhile, Itoh et al. [29] achieved an AUC of 0.956, a sensitivity of 86.7%, and a specificity of 86.7%. Furthermore, Guimarães et al. [34] had 100 normal and 100 abnormal images of pre-cancerous detection during training and 40 normal and 30 abnormal images during testing. Accordingly, he achieved an AUC of 0.981, an accuracy of 92.9%, a sensitivity of 100%, and a specificity of 87.5% via ImageNet. The result was impressive because image samples were acquired from generic databases and were not unique. However, there is a possibility that the databases captured fewer complex conditions of features from atrophic gastritis (AG). In 2018, Nakashima et al. [30] employed GoogLeNet with the Caffe framework to detect *H. pylori* infection that causes AG acquired from 222 enrolled participants. The study showed 105 were *H. pylori*-positive with an AUC reading of 0.97. Aside from this, Zheng et al. [32] achieved an AUC of 0.66, an accuracy of 93.8%, a sensitivity of 91.6%, and a specificity of 98.6%, respectively, via ResNet-50. The study highlighted the identification of AG-infected patients with samples of 1959 patients: 1507 out of 1959 (77%) patients were deemed as training data, 847 (56%) out of 1507 cases were infected with *H. pylori*, and 11,729 gastric images were assigned for training using image augmentation. On the other hand, 452 (23%) out of 1959 patients were deemed as testing data, 310 (69%) out of 452 cases were infected with *H. pylori*, and 3755 gastric images were assigned for testing using image augmentation [32]. It is acknowledged that studies that employ only CNN Inception-V3 score 90.91% accuracy [44]. Similarly, the SVM can achieve high accuracy at 96.3% [37] in identifying gastric cancer.

Subsequently, Alquran et al. [47,48,49] employed ResNet and ShuffleNet Version 2, which are suitable for cervical cytology and cornea ulcer. Nevertheless, gastric cancer has different unique features compared to AG, which are incomparable. Improved recognition methods applied to the detection of *H. pylori*-related AG per se are essential to improve the standards of the diagnostic methods which are currently being used. In addition, further work to detect and upgrade *H. pylori*-related to AG is compelling. The current accuracy stands at 92.9% via ImageNet. As mentioned earlier, however, the samples may be generic and less descriptive since they are public data sets. Furthermore, there is always room for improvement to upgrade the accuracy of AG detection, which currently stands at 93.8% via ResNet-50. Accurate AG detection may promote faster treatment in the early stages of *H. pylori* infection, saving patients from experiencing chronic conditions.

## 3. Materials and Methods

This section elaborates on the feature fusion, feature selection, and classifier employed in the study. The materials and characteristics of atrophic gastritis and normal gastric are presented. The proposed approach, which includes these methods and deep learning specification, is also addressed.

### 3.1. Feature Fusion Canonical Correlation Analysis (CCA)

**Figure 1:**
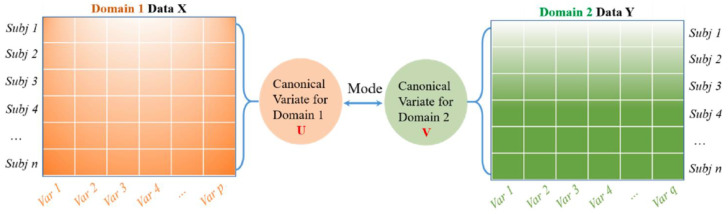
A general schematic for CCA to model the correlation between two feature datasets. Reprinted/adapted with permission frm Ref. [50]. Copyright from Li. et al., Mech. Syst. Signal Process; published by Elsevier Ltd., 2022.

Canonical Correlation Analysis (CCA) is a multivariate-based correlation statistical method. CCA assesses the complex relationships among variable sets that originate from different modalities and is an improved technique for determining the traditional correlation between two sets of variables. Besides this, CCA focuses on a dependence relationship, aiming to model the correlation between variables and the correlation between the two datasets [50]. There are two main concepts to comprehend CCA, which are canonical variables and canonical correlation. Note that canonical variables are linear combinations of the variables in the data sets. Since CCA focuses on correlations between two data sets, users define pairs of canonical variables from the left side of the data set and a second canonical variable from the right side. In the case of a different number of variables in the datasets, it maintains many pairs of canonical variables by determining linear combinations for features with the most correlation in two datasets. On the other hand, Principal Component Analysis (PCA) highlights the determination of linear combinations for the most variance features in one data set. Meanwhile, Multivariate Multiple Regression (MMR) is similar to CCA, whereby the prior method tries to find linear combinations that enable the model of correlations between two data sets. This work uses CCA to extract essential features because its characteristics demonstrate correlated features from two huge datasets [51,52,53]. The idea behind CCA is described in Figure 1.

### 3.2. ReliefF

Relief-F is an instance-based feature selection technique that assesses the feature ability to discriminate between samples rooted in different groups and the similarity between them. Relief-F is a filter-based feature selection method that computes the weighted value of each feature to determine the available essential features [54], as it carries the highest weight. If a feature value differs from its neighboring feature pair of a similar class, the weight decreases. In addition, Relief-F reduces the feature dimension by removing the negative-weighted value feature, which is suitable for a two-class scheme. Correspondingly, it is also useful for multi-class problems [55,56]. Hence, Relief-F was chosen in this work due to its ability to deal with noisy and missing data, a dependence feature that requires swift computation time and is fairly accurate.

### 3.3. Generalized Additive Model (GAM)

A generalized additive model (GAM) is a generalized linear model whereby its response variable depends linearly on unknown smooth functions of some predictor variables, and interest focuses on inference about these smooth functions. Trevor Hastie and Robert Tibshirani developed GAMs to combine the characteristics of generalized linear models with additive models. They can be interpreted as the discriminative generalization of the Naive Bayes generative model. Additionally, GAM is a model that allows the linear model to learn nonlinear relationships. It assumes that instead of using simple weighted sums, it can use the sum of arbitrary functions. Besides, GAMs may overfit when there are too many explanatory variables or a small sample size. For cases where the sample size is too small, the overfitting problem can be solved by increasing the sampling [57]. This work employed GAM due to the ability of the learner to learn nonlinear relationships in the dataset.

### 3.4. Materials

**Figure 2:**
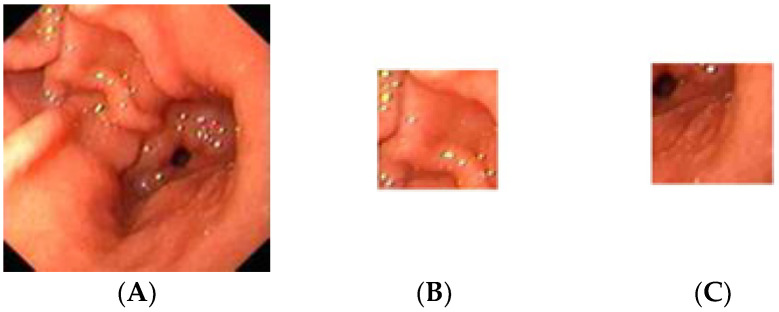
Sample of the normal gastric image cropped into 20 ROIs; (**A**) normal gastric, (**B**) normal ROI_1, (**C**) normal ROI_20.

**Figure 3:**
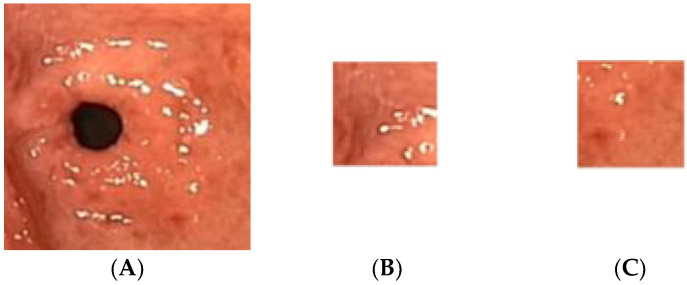
Sample of the atrophic gastric image cropped into 20 ROIs; (**A**) atrophic gastric, (**B**) atrophic ROI_1, (**C**) atrophic ROI_20.

The gastric images were acquired from the Endoscopy Unit, Hospital Universiti Sains Malaysia, Kubang Kerian, Kelantan. The images were captured from 20 patients where 12 were detected as having atrophic gastritis and 8 were classified as normal. The patients had undergone an endoscopy procedure performed by an endoscopy specialist. Each patient’s hardcopy photo 4 by 6 inches in size was obtained from their medical report. The hardcopy photos were scanned in 300 dpi resolution and saved in softcopy form for the study. Thus, 12 images were categorized as atrophic gastritis (AG) and there were 8 normal gastric images. Due to the small-scale number of images available, each image was cropped into 20 regions of interest (ROI) of size 128 × 128 pixels. Therefore, the actual ROIs used in the classification were 244 images from AG and 160 images from normal gastric. The experiment was trained using 70% of the total data and tested on the remaining 30% of the available data. Thus, 282 mixed images of AG and normal gastric were employed for training. On the other hand, 122 mixed normal gastric and AG images were used for testing. Figure 2 illustrates a sample of the normal gastric image, which was then cropped into 20 ROIs. Similarly, Figure 3 shows a sample of the AG image cropped into 20 ROIs.

### 3.5. Characteristics of Normal Gastric and Atrophic Gastric

The human stomach has a shield of mucus lining called the mucosa. The safe lining guards the stomach from the sturdy stomach acid that absorbs food. When the protective lining is damaged, the mucosa becomes inflamed, resulting in gastritis. It is indicative that *Helicobacter pylori* (*H. pylori*) is the most common bacteria that cause gastritis [58].

AG is a histopathological substance characterized by chronic inflammation of the gastric mucosa. The inflamed gastric mucosa experience loss of gastric glandular cells and is reinstated by intestinal-type epithelium, pyloric-type glands, and fibrous tissue. AG is frequently linked with *H. pylori* infection, unidentified environmental factors, and autoimmunity governed in the case of gastric glandular cells. Note that the bacteria disrupt the barrier of mucus that protects the human stomach lining from the acidic juices that help with digestion [58]. Hence, the infection will gradually destroy the cells in the stomach lining if untreated.

### 3.6. Proposed Method

**Figure 4:**
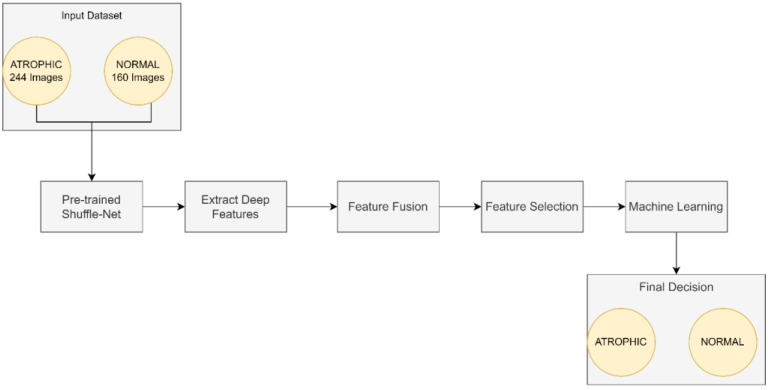
The proposed method for atrophic gastritis detection.

This work focused on the detection of AG versus the normal gut. We highlighted machine learning improvement related to *H. pylori*-related AG infection in the gut. It was observed that previous studies employed GoogLeNet, pre-trained Deep Convolution Neural Networks (DCNN), ImageNet, and ResNet-50. Based on experimentation and analysis, this study used pre-trained ShuffleNet Version 1, CCA feature fusion, Relief-F feature selection method, and GAM classifier, as described in Figure 4.

The pre-trained ShuffleNet used the ReLu layer, which later produced 26,656 features and a global average pooling layer of 544. Subsequently, the high-dimensioned features were processed with feature fusion, known as CCA, resulting in 265 features. The features were further reduced to the 50 most relevant features using the ReliefF feature selection method. Finally, GAM was employed as a machine learner to perform binary classification to identify between atrophic and normal gastritis classes.

### 3.7. Specification of ShuffleNet

This work employed the pre-trained ShuffleNet model already implemented in MATLAB^®^ Version 2021. The pre-trained ShuffleNet utilized the following hyperparameters setting as described in Table 1. It consisted of 171 layers, and employed the root means square propagation (RMSProp) optimizer, a mini-batch size of 64, sixty epochs, and a 0.0004 learning rate.

**Figure 5:**
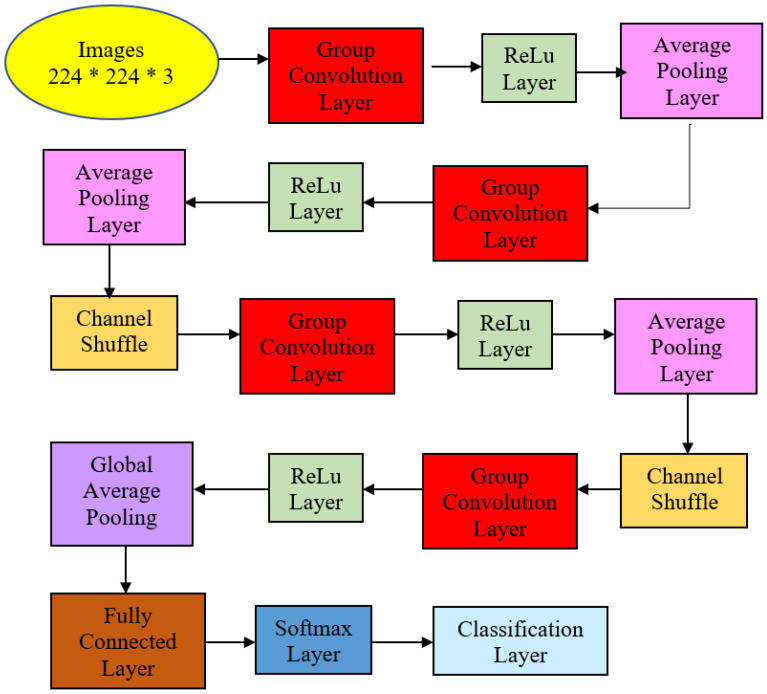
Atrophic Gastritis ShuffleNet structure.

The proposed ShuffleNet structure for AG and its summaries are presented in Figure 5 and Table 2. Table 2 describes the detailed structure of the model. All the images in the dataset were resized to 224 × 224 × 3 pixels to follow the standard image size suggested by ShuffleNet Version 1 network architecture. The model initiated with a group convolution-max pool stem and proceeded with three repeated stages before the finalized global pool-fully connected layer. Consequently, the block began with a 1 × 1 group convolution layer of filter size 112 × 112, kernel size 3 × 3, and stride 2 × 2. Then, the layer was processed via the ReLu layer and followed by an average pooling layer kernel size of 3 × 3 and stride 2 × 2. The initial group convolution did not have a shuffle layer. Thus, the output channel of the next stage was doubled into 56 × 56.

In the next stage, the filter size was 28 × 28 and stride 2 × 2 but not repeated. Subsequently, the average pooling layer featured a 28 × 28 filter size using stride 1 × 2 repeated three times. Finally, ReLu was the activated layer and was followed by batch normalization. Note that ShuffleNet was differentiated from other architectures via shuffle layers as this study had three channels due to colored images. First, the input tensor was reshaped to shuffle the channels from 224 × 224 × 3 channels to 224 × 224 × 3 groups × 3 channels divided by 3 groups. Then, the last two dimensions were permuted, and the input tensor was reshaped to the original shape.

Correspondingly, the next two stages had shuffle layers, in which the filter size in the group convolution layer remained 28 × 28 in Stage 3 with stride 2 × 2 but not repeated. The model applied the ReLu layer and batch normalization at this point. The average pooling layer was of size 14 × 14 with stride 1 × 1, repeated seven times. Then, the channel layers were shuffled via permutation via 3 groups or 3 channels.

Stage 4 featured 7 × 7 maps in the grouped convolution layer with stride 2 × 2 and not repeated. After the layer was activated via ReLu and batch normalization, the average pooling size filter size remained the same: 7 × 7 but this time around with stride 1 × 1 and repeated three times. 

The global average pool layer generated a 1 × 1 feature map with kernel size 7 × 7 that produced the best features, followed by a fully connected layer that applied a softmax layer to proceed with the classification layer with two neurons that depicted classes of normal and AG presence. This structure is described in Figure 5 and Table 2. The transfer learning strategy was performed in the last fully connected layer to be compatible with the number of intended classification classes.

## 4. Results and Discussion

**Figure 6:**
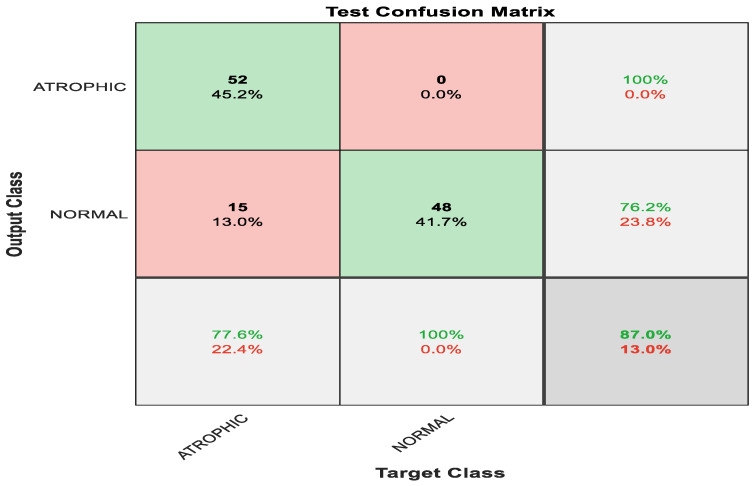
Confusion matrix with results analyzed from the testing data executed via pre-trained ShuffleNet.

Figure 6 shows the confusion matrix with results analyzed from the testing data executed via pre-trained ShuffleNet. Figure 6 demonstrates the results for the binary classes of atrophic gastritis (AG) and normal gut. A total of 52 out of 67 were correctly classified as AG, meaning 77.6% were correctly classified as AG. Whereas the remaining 15 samples were misclassified, meaning 22.4% were misclassified as normal gut. All 48 out of 48 normal gut images were correctly classified, meaning that 100% were classified correctly. The overall accuracy for the pre-trained ShuffleNet was 87.0% for binary classes of AG and normal classes. The misclassification rate for the pre-trained ShuffleNet was 13.0%.

**Figure 7:**
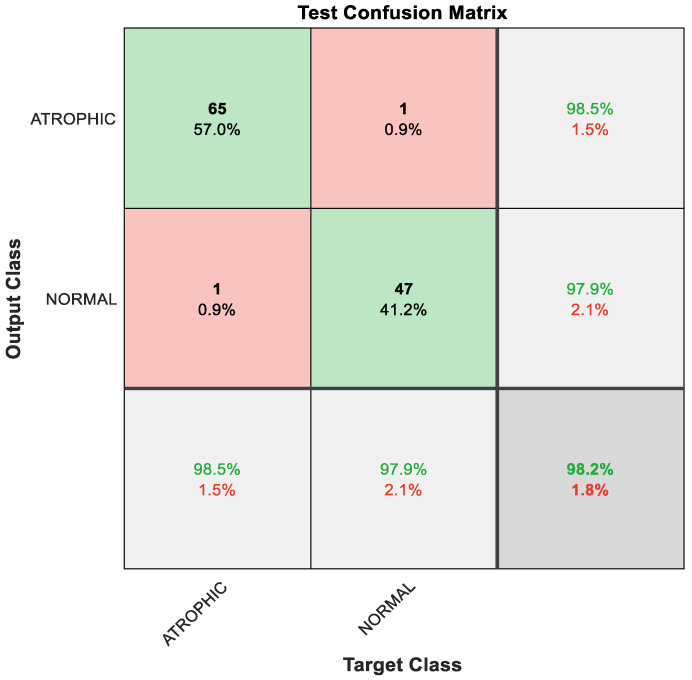
Confusion matrix with results analyzed from the testing data executed via hybrid model and classified by GAM.

The obtained results using deep learning structure were less prominent in distinguishing between atrophic and normal cases. Therefore, a new strategy was proposed to enhance the pre-trained classification result. The proposed method focused on the utilization of feature engineering techniques. The feature engineering enhancement focused on fusion between the ReLU and global average pooling layer. The resultant new features were passed to the feature selection algorithm to nominate the most relevant representative descriptors. Finally, these features were delivered to the generalized additive model (GAM) that distinguished between two classes: atrophic or normal. This hybrid model based on feature engineering clarified the prominent results. Figure 7 shows the confusion matrix with results analyzed from the testing data executed via the hybrid model.

As a result of the techniques that constitute the hybrid model, the classification result improved, with 65 out of 66 instances correctly detected as AG, constituting 98.5% accuracy; the remaining one sample was misclassified, meaning that 1.5% was misclassified as normal gut. Furthermore, 47 out of 48 cases of normal gastric images were accurately identified, i.e., 97.9%; the remaining one sample was misclassified, meaning 2.1% were misclassified as AG. The overall performance of the hybrid model was outstanding at 98.2% accuracy for binary classes of AG and normal classes, as depicted in Figure 7.

Even though the result from the pre-trained ShuffleNet was less prominent, it generated 26,656 significant features via the ReLu layer and 544 significant features from the global average pooling layer, as mentioned in Section 3.6. CCA successfully modeled the relationship between the two data sets of significant features from the ReLu layer and the global average pooling layer respectively. CCA focused on finding linear combinations that most accounted for the correlation of the two datasets. To some extent, ReliefF selected essential features by reducing the feature dimension of the negative-weighted value feature. The features may have consisted of non-linearity characteristics because they were extracted from huge features, thus GAM performed as a better learner. The strength of GAM is that it allows linear modeling to identify the nonlinear relationship among its features.

Table 3 describes the results based on precision and recall, also known as sensitivity, specificity, and accuracy matrices for the pre-trained ShuffleNet and the proposed method. Similarly, Table 4 shows the F1-score results for the mentioned models. Based on the results in Table 4, although the precision and specificity reading of the proposed method compared to the pre-trained network slightly dipped to 98.5% and 97.9% respectively, the recall or sensitivity, accuracy, and F1-score displayed significant improvement in the proposed method. Outside of this, the recall or sensitivity greatly increased from 77.6% in the pre-trained network to 98.5% in the proposed method. Subsequently, accuracy improved from 87.0% in the pre-trained network to 98.2% in the proposed method. Moreover, the F1-score also demonstrated a significant leap from 0.87 in the pre-trained model to 0.98 in the proposed method. These readings indicate that the proposed method can determine true positive cases of AG. Similarly, the proposed method can also predict the cases of atrophic and normal gastric correctly. On a scale from 0 to 1, 1 being the best, the proposed method successfully classified each observation into the correct atrophic and normal classes.

The proposed approach was compared with literature that worked on the same classification problem for endogastric diagnosis images. Table 5 illustrates their methods, type of infection, number of classes, and achievement. The comparison is based on the type of study, of either atrophic gastritis or non-atrophic gastritis. Similar studies of atrophic gastritis were compared based on performance in terms of specificity, sensitivity, and accuracy.

Based on the comparison of our and the existing works in Table 5, some studies [37,40,41,42] were not comparable with our study because their work focussed on either gastric cancer or determining the location of infection. Furthermore, some studies [30,31,33] had insufficient information related to the results presented here and thus were incomparable to this work. It is evident from Table 5 that study [28] achieved specificity, sensitivity, and accuracy in the range of less than 83.5%. Meanwhile, study [44] achieved much a better result compared to [28]; however the specificity, sensitivity, and accuracy were much lower than 91.5%; thus this work was insignificant to this research, which achieved specificity, sensitivity, and accuracy all above 97%. References [28,44] achieved a less significant result compared to this work because they employed GoogleNet with Inception Module, which did not employ group convolution and had fixed convolution sizes for each layer, thus missing significant features. On the other hand, the result of this study is much better because it employed ShuffeNet, which performed group convolution and each output channel related to the input channels outside the group. As for study [34], even though their sensitivity reading was 100% compared to the this study’s sensitivity reading of 98.5%, its specificity was 87.5% and its accuracy was 92.9%, both much lower than the readings in this study. Unlike this in this study, it is suggested that study [34], which employed ImageNet, was actually a large-scale dataset rather than deep learning network itself. Study [32], which employed ResNet-50, achieved a slightly better specificity of 98.6% compared to that of this work with its specificity reading of 98.5%. Nevertheless, study [32]’s sensitivity reading of 91.6% and accuracy reading of 93.8% were both much lower than this study’s readings of 98.5% and 98.2% respectively. Unlike in this study, study [32], which employed ResNet-50, suffered a degradation problem when the network became deeper. On the other hand, study [29], which employed GoogleNet, achieved a specificity of 98.5%, slightly better than the 97.9% reading in this study. Nevertheless, study [32]’s sensitivity and accuracy fell short at 96.9% and were not applicable respectively compared to the readings in this study. This study’s sensitivity reading was at 98.5% and its accuracy was at 98.2%. It is evident this study’s accuracy, at 98.2%, was higher than studies [28,32,34,44], which all had readings are lower than 94%.

Compared to existing works, this study had the best accuracy because it employed a hybrid model that included pre-trained ShuffleNet. The pre-trained ShuffleNet performed group convolution and each output channel was related to the input channels outside the group due to the shuffle mechanism. This mechanism strengthened the blocks of information representation, thus generating essential features. In the hybrid model, the essential features were channeled to perform a feature engineering technique besides learning with GAM. The feature engineering technique was supported by the Canonical Correlation Analysis (CCA) feature fusion approach and ReliefF feature selection method that successfully extracted relevant and essential features before the finalized classification method was executed. Indeed, CCA was supported in one of the stages whereby the method took a linear combinations approach that enabled the model correlation between two feature datasets. Furthermore, during the classification process, GAM successfully learned nonlinear relationships in the dataset and managed the small sample size problem by generating many regions of interest (ROIs).

## 5. Conclusions

To sum up, some previous studies have highlighted the detection of gastric cancer, yet studies to detect atrophic gastritis (AG) are equally important. This is because *Helicobacter pylori* (*H. pylori*)-related AG is the initial indicator of problematic gut condition. Furthermore, undetected and untreated AG may worsen into a chronic condition and eventually develop into gastric cancer.

This study proposed a hybrid model which included a pre-trained ShuffleNet that shuffled channels in a group-wise manner. The pre-trained ShuffleNet employed a cheap approach that later produced 26,656 features from the ReLu layer and 544 rich features from the global average pooling layer. Although accuracy at the pre-trained ShuffleNet stage was 87% and less prone to achieve an ideal model with an F1-score reading of 0.87, the rich and essential features were successfully identified. Extended approaches generated more relevant features from the deep features; namely feature fusion Canonical Correlation Analysis (CCA), which produced 265 features, and ReliefF feature selection, which generated 50 relevant features from the deep features. This was evident from the huge improvement to 98.2% accuracy and an almost ideal model based on an F1-score value of 0.98.

As mentioned earlier, the proposed hybrid model inclusive of pre-trained ShuffleNet achieved an accuracy of 98.2%, which was higher than similar studies that attempted to detect atrophic gastritis in binary classes of atrophic gastritis versus normal gastric condition. Unlike the method proposed in this study, some of the existing works employed a basic deep learning approach without an extended approach, which resulted in much lower accuracy and fewer chances to create an ideal model to detect atrophic gastritis or normal gastric condition.

Through endless efforts by researchers in medicine and artificial intelligence (AI) via the proposed hybrid model, the physician can detect the occurrence of AG as an initial step to screen the *H. pylori* infection in the human stomach, thus reducing the possibility of a further chronic condition in the gastric.

## Figures and Tables

**Table 1:** Pre-trained ShuffleNet hyperparameters setting.

Hyperparameters Setting	
Optimizer	RMSprop
Number of Layers	171
Mini batch size	64
Maximum Epoch	60
Learning Rate	0.0004

**Table 2:** Structure summaries for Atrophic Gastritis ShuffleNet.

Layer	Information
Input Layer	Size: 224 × 224 × 3
	Number of Filters: 112
Group Conv1	Kernel Size: 3 × 3
	Stride: 2 × 2
	Padding: 0
Activation Layer	ReLu
	Type: Average Pooling
Pooling Layer	Kernel Size: 3 × 3
	Stride: 2 × 2
	Padding: 0
	Number of Groups: 3
Group Convolution Layer	Number of Filters: 56
	Kernel Size: 3 × 3
	Padding: 0
Activation Layer	ReLu
	Type: Average Pooling
Pooling Layer	Kernel Size: 3 × 3
	Stride: 1 × 1
	Padding: 0
	Shuffle layer
Group Convolution Layer	Number of Filters: 28
	Kernel Size: 3 × 3
	Stride: 1 × 1
	Padding: 0
Activation Layer	ReLu
	Shuffle layer
	Number of Groups: 3
Group Convolution Layer	Number of Filters: 11
	Kernel Size: 3 × 3
	Stride: 1 × 1
	Padding: 0
Activation Layer	ReLu
	Type: Global Average Pooling
Fully Connected Layer	2 neurons
Softmax Layer	
Classification Layer	

**Table 3:** Results based on precision, recall, specificity, and accuracy matrices for the pre-trained ShuffleNet and proposed method.

	Pre-Trained ShuffleNet (%)	Proposed Method (%)
Precision	100.0	98.5
Recall = Sensitivity	77.6	98.5
Specificity	100.0	97.9
Accuracy	87.0	98.2

**Table 4:** F1-score result for the pre-trained ShuffleNet and proposed method.

	Pre-Trained ShuffleNet	Proposed Method
FI-score	0.87	0.98

**Table 5:** Comparison of the proposed approach with literature on similar endogastric diagnosis classification problems.

Study	Method	Type	Classes	Accuracy
Shichijo S. et al. (2017) [28]	Deep CNN, 22 layers, pre-trained GoogLeNet via ImageNet	*H. pylori*	2	AUC = 0.89, Specificity = 81.9%, Sensitivity = 83.4%, Accuracy = 83.1%
Takiyama H. et al. (2018) [40]	GoogLeNet	Anatomical location of the upper digestive tract		AUC = 0.99, Specificity = 98.5%, Sensitivity = 96.9%, Accuracy = N/A
Itoh et al. (2018) [29]	GoogLeNet	*H. pylori*	2	AUC = 0.956, Specificity = 86.7%, Sensitivity = 86.7%, Accuracy = N/A
Hirasawa et al. (2018) [42]	SSD	Gastric cancer	2	AUC = N/A, Specificity = N/A, Sensitivity = 92.2%, Accuracy = N/A
Kanesaka et al. (2018) [37]	SVM	Early gastric cancer	2	AUC = N/A, Specificity = 95%, Sensitivity = 96.7%, Accuracy = 96.3
Nakashima et al. (2018) [30]	GoogLeNet; Caffe	*H. pylori*	2	AUC = 0.97, Specificity = N/A, Sensitivity = NA, Accuracy = NA,
Shichijo S. et al. (2019) [31]	Pre-trained DCNN	*H. pylori*	2	Accuracy = 80% (negative), 84% (eradicated), 48% (positive)
Dong-hyun et al. (2019) [33]	GoogLeNet; Inception module	Gastric disease	2	ROC = 0.85 (normal) and 0.87 (abnormal)
Zheng et al. (2019) [32]	ResNet-50	*H. pylori*	2	AUC = 0.66, Specificity = 98.6%, Sensitivity = 91.6%, Accuracy = 93.8
Wu et al. (2019) [41]	VGG ResNet	26 locations of gastric cancer		AUC = N/A, Acc = 90, Sensitivity = N/A, Specificity = N/A
Ishioka et al. (2019) [43]	SSD	Gastric cancer	2	AUC = N/A, Specificity = N/A, Sensitivity = 94.1%, Accuracy = N/A
Horiuchi et al. (2020) [45]	GoogLeNet	Gastric cancer vs. gastritis	2	AUC = 0.85, Specificity = 71.0%, Sensitivity= 95.4%, Accuracy= 85.3%
Zhu et al. (2019) [46]	ResNet	Gastric cancer	2	AUC = 0.94, Specificity = 95.56%, Sensitivity= 76.47%, Accuracy= 89.16%
Guimarães P et al. (2020) [34]	ImageNET	*H. pylori*	2	AUC = 0.981, Specificity = 87.5%, Sensitivity = 100%, Accuracy = 92.9%
Li et al. (2020) [44]	Inception-V3	*H. pylori*	2	AUC = N/A, Specificity = 90.64%, Sensitivity = 91.18%, Accuracy = 90.9%
Our study	Pre-trained CNN: ShuffleNet version 1	*H. pylori*	2	Test: 98.2%; Atrophic sensitivity (98.5%), Normal sensitivity (97.9%)

## Data Availability

Not applicable.
